# The DataHarmonizer: a tool for faster data harmonization, validation, aggregation and analysis of pathogen genomics contextual information

**DOI:** 10.1099/mgen.0.000908

**Published:** 2023-01-23

**Authors:** Ivan S. Gill, Emma J. Griffiths, Damion Dooley, Rhiannon Cameron, Sarah Savić Kallesøe, Nithu Sara John, Anoosha Sehar, Gurinder Gosal, David Alexander, Madison Chapel, Matthew A. Croxen, Benjamin Delisle, Rachelle Di Tullio, Daniel Gaston, Ana Duggan, Jennifer L. Guthrie, Mark Horsman, Esha Joshi, Levon Kearny, Natalie Knox, Lynette Lau, Jason J. LeBlanc, Vincent Li, Pierre Lyons, Keith MacKenzie, Andrew G. McArthur, Emily M. Panousis, John Palmer, Natalie Prystajecky, Kerri N. Smith, Jennifer Tanner, Christopher Townend, Andrea Tyler, Gary Van Domselaar, William W. L. Hsiao

**Affiliations:** ^1^​ University of British Columbia, Vancouver, BC, Canada; ^2^​ Faculty of Health Sciences, Simon Fraser University, Burnaby, BC, Canada; ^3^​ Cadham Provincial Laboratory, Winnipeg, MB, Canada; ^4^​ National Microbiology Laboratory, Public Health Agency of Canada, Winnipeg, MB, Canada; ^5^​ Alberta Precision Labs, Edmonton, AB, Canada; ^6^​ Department of Laboratory Medicine and Pathology, University of Alberta, Edmonton, AB, Canada; ^7^​ Laboratoire de santé publique du Québec, Montréal, QC, Canada; ^8^​ Public Health Ontario Laboratory, Toronto, ON, Canada; ^9^​ Department of Pathology and Laboratory Medicine, Nova Scotia Health, Halifax, NS, Canada; ^10^​ The Hospital for Sick Children, Toronto, ON, Canada; ^11^​ Public Health Agency of Canada, Moncton, NB, Canada; ^12^​ Roy Romanow Provincial Laboratory, Regina, SK, Canada; ^13^​ Michael G. DeGroote Institute for Infectious Disease Research & Department of Biochemistry and Biomedical Sciences, McMaster University, Hamilton, ON, Canada; ^14^​ BCCDC Public Health Laboratory, Vancouver, BC, Canada; ^15^​ Eastern Health, St. John’s, NL, Canada

**Keywords:** contextual data, data management, genomic surveillance, harmonization, metadata

## Abstract

Pathogen genomics is a critical tool for public health surveillance, infection control, outbreak investigations as well as research. In order to make use of pathogen genomics data, they must be interpreted using contextual data (metadata). Contextual data include sample metadata, laboratory methods, patient demographics, clinical outcomes and epidemiological information. However, the variability in how contextual information is captured by different authorities and how it is encoded in different databases poses challenges for data interpretation, integration and their use/re-use. The DataHarmonizer is a template-driven spreadsheet application for harmonizing, validating and transforming genomics contextual data into submission-ready formats for public or private repositories. The tool’s web browser-based JavaScript environment enables validation and its offline functionality and local installation increases data security. The DataHarmonizer was developed to address the data sharing needs that arose during the COVID-19 pandemic, and was used by members of the Canadian COVID Genomics Network (CanCOGeN) to harmonize SARS-CoV-2 contextual data for national surveillance and for public repository submission. In order to support coordination of international surveillance efforts, we have partnered with the Public Health Alliance for Genomic Epidemiology to also provide a template conforming to its SARS-CoV-2 contextual data specification for use worldwide. Templates are also being developed for One Health and foodborne pathogens. Overall, the DataHarmonizer tool improves the effectiveness and fidelity of contextual data capture as well as its subsequent usability. Harmonization of contextual information across authorities, platforms and systems globally improves interoperability and reusability of data for concerted public health and research initiatives to fight the current pandemic and future public health emergencies. While initially developed for the COVID-19 pandemic, its expansion to other data management applications and pathogens is already underway.

## Significance as a BioResource to the community

Contextual data, also known as ‘metadata’, are critical for interpreting sequence data, answering biological questions and informing decision-making during public health emergencies. The DataHarmonizer is a data management tool for harmonizing and validating pathogen genomics contextual data according to different data standards and specifications, and transforming genomics contextual data into submission-ready formats required for private databases [e.g. Public Health Agency of Canada (PHAC) National SARS-CoV-2 genomic sequence database] or public repositories [e.g. Global Initiative on Sharing All Influenza Data (GISAID), National Center for Biotechnology Information (NCBI), CanCOGeN VirusSeq Data Portal]. The tool saves time and resources via automated error detection and batch editing, and improves consistency and interoperability of contextual data via validation. The standardized output future-proofs data for different downstream analyses and better enables the information to be used by organizations worldwide. The DataHarmonizer has been used by public health organizations and research institutions across Canada as part of the COVID-19 pandemic response, and is increasingly being used in a number of other applications.

## Data Summary

1. The DataHarmonizer is freely available at https://github.com/cidgoh/DataHarmonizer [last accessed 2 December 2021].
2. Technical details regarding template design can be found at https://github.com/cidgoh/DataHarmonizer/wiki/DataHarmonizer-Templates [last accessed 2 December 2021].
3. Canadian SARS-CoV-2 contextual data harmonized using The DataHarmonizer can be found in the NCBI Umbrella BioProject PRJNA623807 and the CanCOGeN VirusSeq Data Portal (https://virusseq-dataportal.ca/) [last accessed 2 December 2021].
4. The data standard implemented by The DataHarmonizer can be found at https://github.com/pha4ge/SARS-CoV-2-Contextual-Data-Specification [last accessed 2 December 2021].

## Introduction

Tracking the spread and evolution of the SARS-CoV-2 virus at global, national and regional scales has been aided by the analysis of genomic sequence and epidemiological data [1–7]. SARS-CoV-2 genomics contextual data, also known as genomic sequence ‘metadata’, include laboratory [e.g. date and location of testing, cycle threshold (Ct) values], clinical (e.g. hospitalization status, outcomes), epidemiological (e.g. age, gender, exposures, travel history) and methodological (e.g. sampling strategy, sequencing, bioinformatics) information, and is critical for interpreting viral sequence data, answering biological questions and informing decision-making during public health emergencies.

Contextual data collected by academic, hospital and public health laboratories are often inconsistent across institutions and jurisdictions due to variations in information management systems, data collection practices and the lack of data standards [8, 9]. The variability in data collection instruments and practices creates data silos and complicates data sharing and integration, hindering public health investigations. Variability in local data collection also propagates inconsistencies to public repositories when data are shared [10]. Contextual data that are structured, consistent and compliant with data standards are easier to understand and process, and can be more easily aggregated and reused for different downstream analyses [11, 12]. However, there is a paucity of open source data management tools that enable the implementation of data standards in public health settings, which ultimately results in reduced uptake and use of standards.

To improve the consistency, interoperability and reusability of contextual data across different data providers and sources, we have developed the DataHarmonizer, a spreadsheet application that implements SARS-CoV2 data standards [i.e. the Canadian COVID-19 Genomics Network (CanCOGeN), Public Health Alliance for Genomic Epidemiology (PHA4GE)] via data management templates. The DataHarmonizer enables curators to enter and validate data through a graphical user interface and then transform data entries into a variety of repository submission-ready formats [e.g. GISAID; NCBI BioSample, Sequence Read Archive (SRA), or GenBank databases (NCBI); the CanCOGeN VirusSeq Data Portal, the PHAC National Microbiology Laboratory (NML) Laboratory Information Management System (LIMS); and the Canadian Network for Public Health Intelligence Portal (CNPHI)] to support data sharing. The DataHarmonizer is implemented in a browser-based JavaScript environment, HTML and CSS environment, and is supported on all modern versions of Google Chrome (version 49+), Microsoft Edge (version 12+) and Mozilla Firefox (version 34+). The DataHarmonizer is freely available for download at https://github.com/cidgoh/DataHarmonizer/ under an MIT open-source licence.

## Theory and implementation

### Data standards, templates and user guides

Currently, the DataHarmonizer offers two SARS-CoV-2 contextual data collection templates based on the specification co-developed by our team at Simon Fraser University’s Centre for Infectious Disease Genomics and One Health (CIDGOH) (https://www.cidgoh.ca) and the Public Health Alliance for Genomic Epidemiology (PHA4GE) (https://pha4ge.org [13]). The first template implements the PHA4GE standard, which is designed for international use, and the second template is a customized version of the same specification designed for CanCOGeN. Both templates offer standardized fields, data formats and pick lists of controlled vocabulary to structure information pertaining to sample identifiers and accession numbers, sample collection and sampling strategy, host information, host exposure and re-infection data, host vaccination details, lineage/variant information, pathogen diagnostic testing, and sequencing/bioinformatic methods. Calendar widgets facilitate date entries in ISO 8601 format. Fields and controlled vocabulary in both templates were improved iteratively based on regular use and feedback by stakeholders and updated to reflect the evolution of the pandemic. Templates are versioned on GitHub using nomenclature modelled on semantic versioning.

**Fig. 1.**
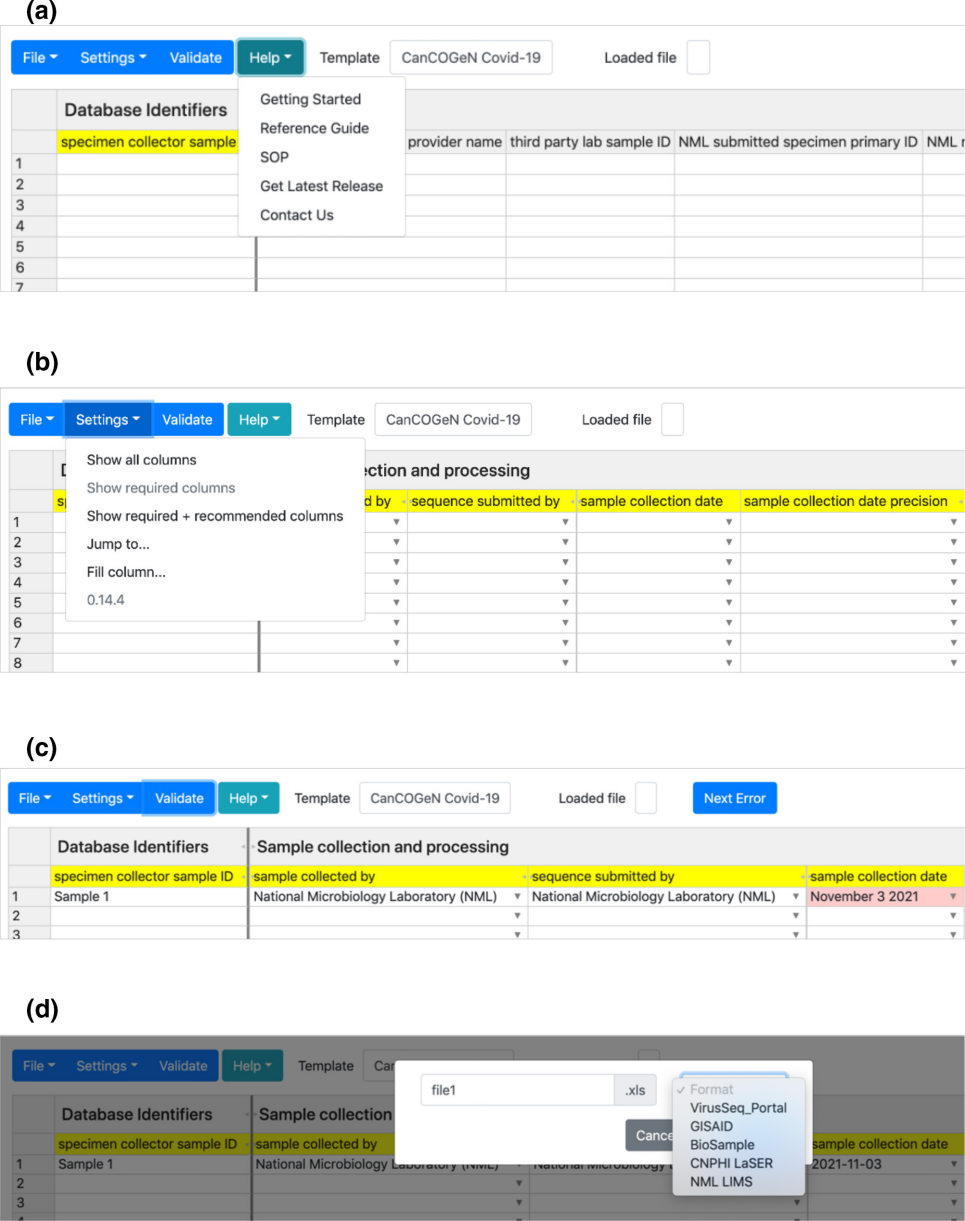
The DataHarmonizer interface. (**a**) Users can access instructional videos, the reference guide and protocols for curation. Data providers can also access field-level guidance by double clicking on field headers. (**b**) Fields are colour-coded to indicate those that are required for Canadian SARS-CoV-2 surveillance (yellow), recommended (purple) and optional (grey). Users access different features, such as toggling between fields and automated filling of columns, via the control panel. (**c**) Validation of data highlights missing required information as well as errors which are highlighted in red. The ‘Next Error’ button enables users to scroll through and resolve errors systematically. (**d**) Users can export their data in different submission-ready formats.

**Fig. 2.**
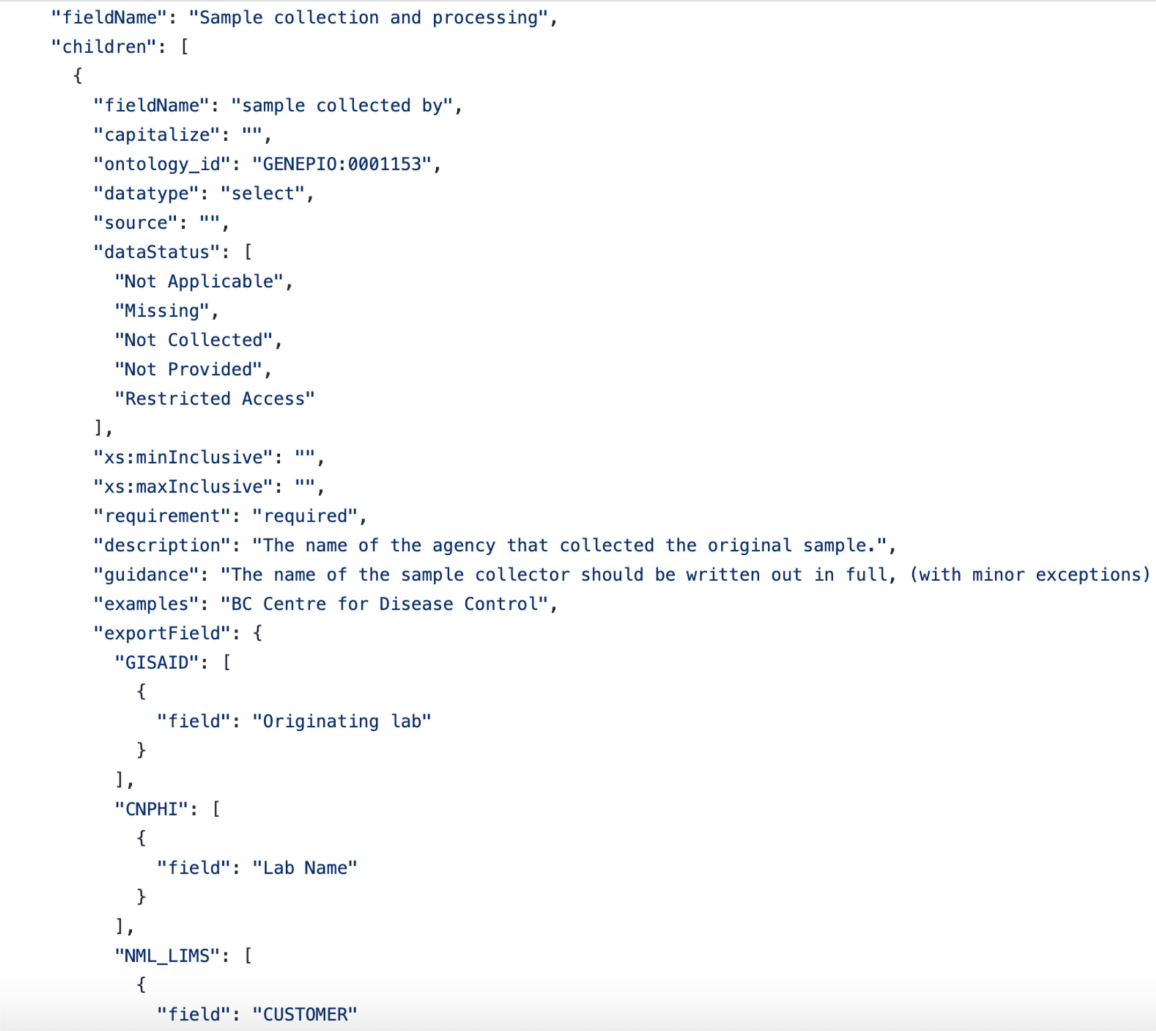
Excerpt of a JSON file used to dynamically generate The DataHarmonizer application interface and functionality.

Users can change templates by exploring the File tab, clicking ‘Change template’, followed by selecting the desired template from the pulldown menu (Fig. 1). Data management templates (containing column headers, descriptions, formatting requirements and standardized vocabularies) are stored as JSON files and used to dynamically generate the application interface and its functionality using the JavaScript grid table generator Handsontable (9.0.2). The separation of the data structure from the code used to generate the application interface makes the DataHarmonizer system modular, and enables the functionality of the DataHarmonizer to be easily extended to a variety of use cases using different data management templates without requiring significant edits to the rest of the application (Fig. 2). Detail outlining the JSON specification for templates is available at https://github.com/cidgoh/DataHarmonizer/wiki/DataHarmonizer-Templates. Guidance for software developers creating project-specific templates is available at the same location. Each template contains specifications for field standardization and validation, and allows export to desired formats, with optional customized code in an export .js file to handle irregular conversion rules.

To support contextual data standardization and curation, fields prioritized for surveillance are colour-coded in yellow (e.g. geographical location of sampling and date fields), fields that are recommended (e.g. methods) are colour-coded in purple and optional fields are colour-coded in grey (Fig. 1). User guides and training materials accompany the tool (https://github.com/cidgoh/DataHarmonizer/wiki/DataHarmonizer-Template-SOPs). Users can double-click template headers to see the definition of each field, guidance for how to populate it and examples of usage. The data dictionary is also available in the field-level Reference Guide available from the ‘Help’ tab. Reference guides based on information stored in other project-specific templates can also be automatically generated. Also available under the ‘Help’ tab is a series of short instructional videos to orient first-time users, as well as a curation standard operating procedure (SOP) outlining practical, ethical and privacy considerations for sharing public health data. Longer training videos are also available at https://github.com/cidgoh/DataHarmonizer/wiki/CanCOGeN-Contextual-Data-Specification.

### Data entry, validation and transformation

Users can enter data directly into the spreadsheet-based interface, upload a spreadsheet compatible with the data specification or copy/paste data from other applications (e.g. Microsoft Excel and Google Sheets). There are a number of curation features to help improve data entry under the ‘Settings’ tab, such as ‘Jump to’, ‘Fill Column’ and ‘Show required fields’ (Fig. 1, Table 1).

**Table 1.** Summary of the features and functionality of The DataHarmonizer

Tab	Feature	Description
File	Export to	Enables the user to automate transformation of standardized data into multiple downstream formats at the click of a button. Formats include: GISAID NCBI BioSample NCBI SRA NCBI GenBank NCBI GenBank-source-modifiers VirusSeq Data Portal NML LIMS
File	Change template	Enables the user to select initiative-specific templates (e.g. CanCOGeN, PHA4GE, other draft templates).
Settings	Show required fields	Enables the user to display only ‘required’ fields (colour-coded yellow)
Settings	Jump to	The Jump to function enables users to move quickly across the template to find the fields they need
Settings	Fill column	Automates filling columns with the same data for all samples
Validate	Next Error	Upon validation, errors or missing information will be highlighted in red. Users can systematically address errors by using the ‘Next Error’ button which only appears in the top right of the interface when there are errors. The ‘Next Error’ button disappears when all errors have been corrected
Help	Getting started	Provides a carousel of short videos demonstrating how to use the various functions and features
Help	Reference guide	Definitions of fields, guidance for entering data, examples of use.
Help	SOP	Instructions for installation, as well as entering and curating data. The SOP provides examples of how to enter descriptions of different sample types

**Fig. 3.**
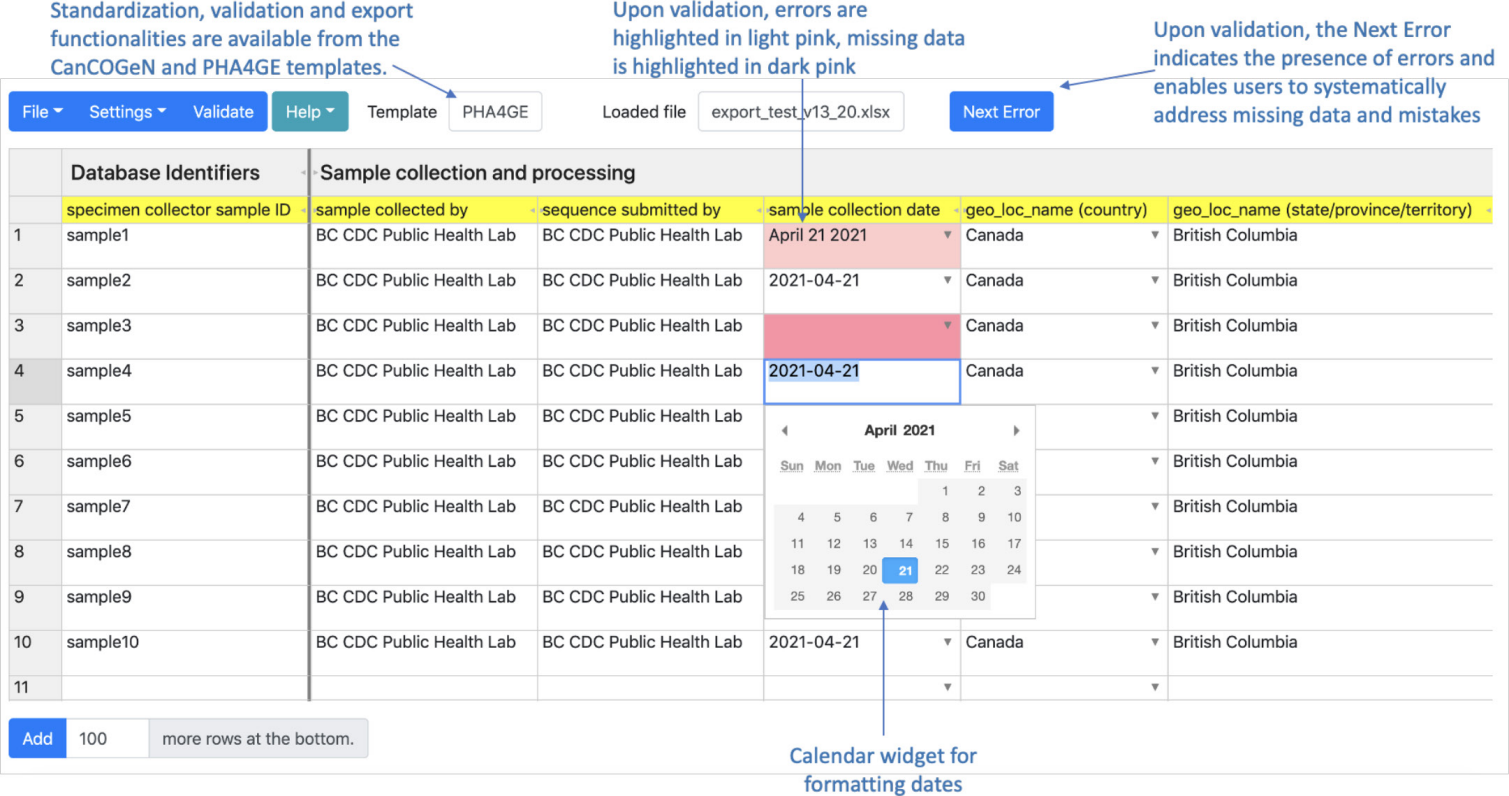
Validation of data. Missing or incorrect values are highlighted to better direct curation efforts. Curators can systematically address errors using the ‘Next Error’ button, which disappears when all issues have been addressed.

Clicking the ‘Validate’ button will highlight cells with spelling mistakes, formatting errors and missing required values (Fig. 3). The ‘Next Error’ button enables users to toggle between highlighted cells to correct errors. The button disappears when all errors have been addressed. The version of the DataHarmonizer used to perform curation is captured upon validation in the ‘DataHarmonizer Provenance’ field.

**Fig. 4.**
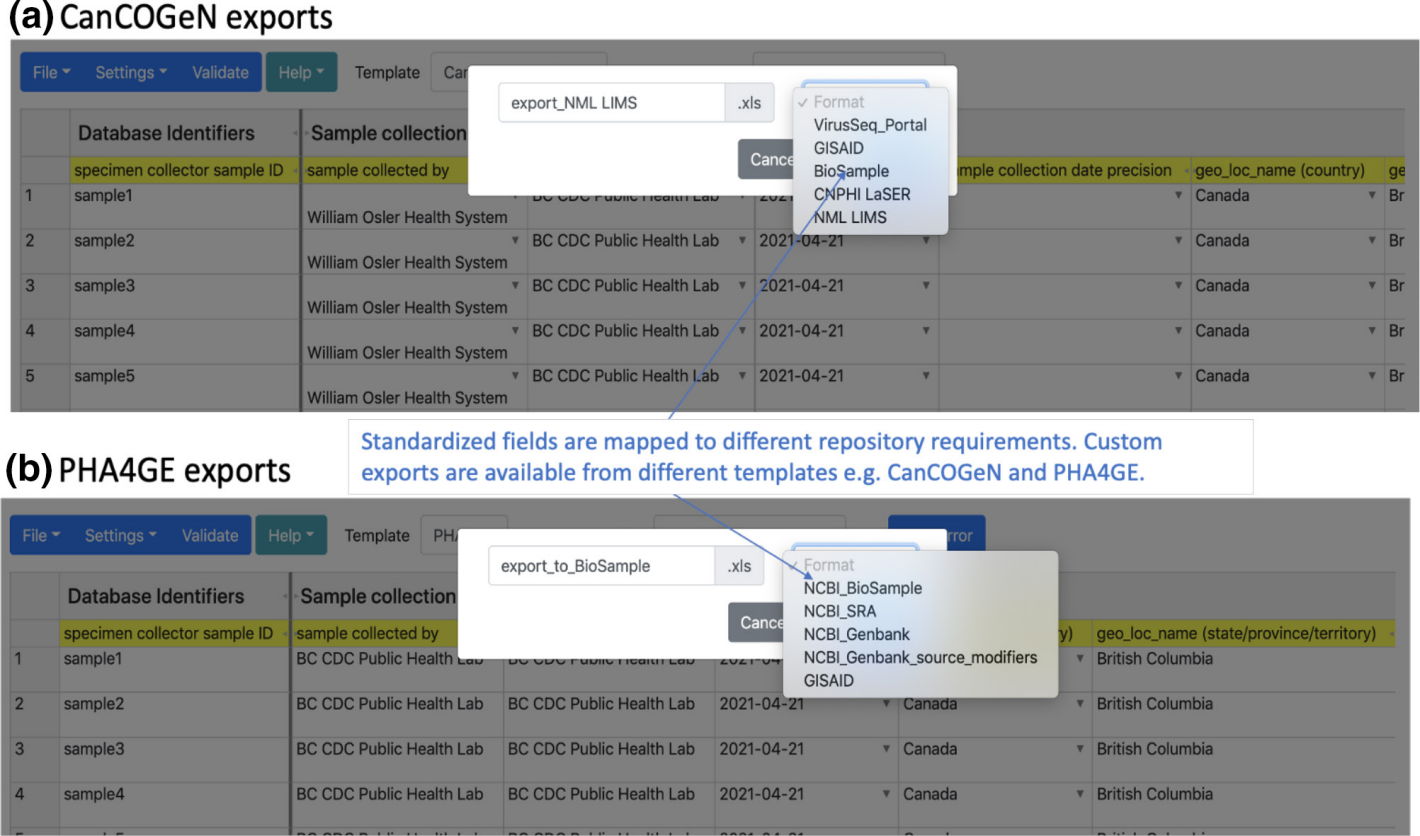
Customized exports automate data transformation for submission to a variety of third-party databases. (**a**) For Canadian national surveillance, contextual data can be exported to Canadian-specific portals and databases (e.g. VirusSeq Portal, CNPHI LaSER and NML LIMS) as well as international repositories (e.g. GISAID, NCBI). (**b**) Export from the PHA4GE template enables formatting for international databases.

Different public repositories and databases have different submission requirements (fields, formats, etc.). Transforming contextual data for different submissions can be very time consuming, and often the requirements are unclear to data providers. To reduce the burden of preparing different submissions, the DataHarmonizer enables automated transformations for international repositories (e.g. GISAID, NCBI BioSample, SRA and GenBank) as well as Canadian portals and databases (e.g. CNPHI, NML LIMS, VirusSeq Data Portal) (Fig. 4) . Entered data can be exported and transformed by clicking the File tab, selecting the ‘Export to’ option followed by the desired format from the pick list. Exports can then be submitted to GISAID and the International Nucleotide Sequence Database Collaboration (specifically NCBI) repositories using PHA4GE protocols (https://www.protocols.io/workspaces/pha4ge), and to Canadian databases via CanCOGeN protocols.

The DataHarmonizer is scalable and can process large datasets quickly. For example, benchmarking tests (MacBook Pro with a four-core CPU and 16 GB of RAM) demonstrated that a dataset of 45 000 records with at least one error engineered into each record took 12 s to validate, and 20 s to export to NML-LIMS format. Automated data entry functionality like the ‘Fill Column’ feature took 6–15 s to process, and the file took 28 s to open.

### Operational implementation and use cases

The DataHarmonizer is being used to harmonize Canadian SARS-CoV-2 contextual data used for national genomic surveillance. Canada has a decentralized health system. As health care is a matter under provincial jurisdiction, each of the ten Canadian provinces, three territories and Indigenous authorities governs and manages its own data collection and stewardship, as well as privacy legislation and practices. In the CanCOGeN viral genomic sequencing initiative (VirusSeq), each province/territory and their partner research institutes have their own installation of the DataHarmonizer, which is used to harmonize contextual data before submission to the national database housed at the NML (Winnipeg, MB). Provincial/Territorial/institutional data curators worked with the development team over the course of the pandemic to provide feedback, request feature and vocabulary updates, and test performance improvements. Examples of harmonized contextual data also submitted to the NCBI BioSample database are provided (Table 2). The DataHarmonizer has been used to curate and transform hundreds of thousands of Canadian SARS-CoV-2 contextual data records during the pandemic.

**Table 2.** Examples of Canadian SARS-CoV-2 NCBI BioSamples harmonized using The DataHarmonizer While the fields are specified by allowed NCBI attribute packages, the values that go into those fields are harmonized and validated using the DataHarmonizer according to the data standard created in collaboration with PHA4GE.

Data provider	BioSample accession
Laboratoire de santé publique du Québec (LSPQ/INSPQ)	SAMN20419171
Toronto Invasive Bacterial Diseases Network/McMaster University	SAMN17505491
New Brunswick Diagnostic Virology Reference Center/Public Health Agency of Canada (National Microbiology Laboratory)	SAMN16784833
Cadham Provincial Laboratory/Public Health Agency of Canada (National Microbiology Laboratory)	SAMN16689906

The DataHarmonizer is also being adapted for inter-agency harmonization of foodborne pathogen contextual data for a Canadian One Health genomics initiative studying antimicrobial resistance in the food and food production environments [Antimicrobial Resistance Genomics Research and Development Initiative (AMR-GRDI); https://grdi.canada.ca/en/projects/antimicrobial-resistance-amr]. In addition to a draft template developed to structure information according to a multi-agency Canadian One Health data specification, the DataHarmonizer also supports agency-specific templates to facilitate exports from agency databases according to the national standard (i.e. GRDI and the PHAC-specific *Dexa* data management system).

Owing to its extensibility, additional templates can be developed to enable standardized data entry, harmonization and transformation. PHAC has already created a new template specific to their needs for Canadian SARS-CoV-2 international border testing, which is privately hosted on their local installations of the DataHarmonizer. The US Department of Energy’s National Microbiome Data Collaborative (NMDC) is developing an open-access framework that facilitates more efficient use of microbiome data for applications in energy, environment, health and agriculture. The NMDC is currently developing MIxS DataHarmonizer templates for its contextual data harmonization needs (https://github.com/turbomam/DataHarmonizer). Future development priorities include adding login capability for users to use DataHarmonizer as a front-end to server-managed datasets. We welcome new partners with their own use cases to extend the functionalities of the DataHarmonizer.

### Installation and availability

The DataHarmonizer is open source and freely available from GitHub (https://github.com/cidgoh/DataHarmonizer/releases) under an MIT software licence. The tool can run completely locally by downloading the ‘Source code’ zip file and clicking on the ‘main.html’ file, which launches the application. Its lightweight installation enables users to update their instance easily when new features and templates are developed. The DataHarmonizer is built for offline use, sourcing all code generation libraries locally, which increases data security and privacy relative to online spreadsheet applications (e.g. Google Sheets), which some public health authorities are prevented from using for security or privacy reasons.

## Discussion

Data standards are important for increasing consistency and interoperability of contextual data across databases and platforms. Data management tools implementing these standards are required to promote real-time uptake of the standards in public health organizations. The DataHarmonizer is an easy-to-use, lightweight application specifically designed to harmonize and validate contextual information according to community standards, such as the PHA4GE SARS-CoV-2 contextual data specification. Other data management tools with overlapping functionality include the Office of Cyber Infrastructure and Computational Biology (OCICB) and the National Institute of Allergy and Infectious Diseases (NIAID)’s METAGENOTE, REDCap [14, 15], and the command-line transformation tool multiSub (https://github.com/maximilianh/multiSub). Like the DataHarmonizer, both METAGENOTE and REDCap facilitate the production of standardized data collection templates with validation functionality. However, the DataHarmonizer improves upon these existing technologies in two critical aspects. Its offline operation is a key data security feature as many public health organizations do not allow the raw data to leave their networks for processing in an online environment. The DataHarmonizer also allows users to validate data at anytime during editing, unlike METAGENOTE. Furthermore, the DataHarmonizer’s spreadsheet interface provides a simpler user-editing experience, allowing records to be quickly copied/pasted into the application, unlike REDCap. The multiSub tool is useful for transforming datasets between public repository submission requirements but does not align data values to data standards. While the DataHarmonizer offers many advantages to users, we encourage the uptake of all these tools, as well as data management best practices, for example making data Findable, Accessible, Interoperable, Reuseable (FAIR) [16], and more robust data sharing frameworks to ensure the best quality contextual data accompanies sequence data.

The larger contextual data ecosystem is complex as there are many moving parts, such as public and organization-specific databases, local data management tools, as well as publicly available services. The controlled vocabulary developed for SARS-CoV-2 in the DataHarmonizer templates has been made available to the public health and research communities via different OBO Foundry ontologies [e.g. the Genomic Epidemiology Ontology (GenEpiO)] [13]. The standardized fields and terms are also discoverable via various ontology look-up services such as EMBL-EBI’s OLS (https://www.ebi.ac.uk/ols/index) as well as online text matching services such as EMBL-EBI’s ZOOMA (https://www.ebi.ac.uk/spot/zooma/), which make use of those ontologies. It should be noted that initially aligning variable public health data to any standard upstream of these data management tools is a persistent challenge. Text-mining and text-matching tools such as LexMapr (developed by the authors) and ZOOMA can help lower these upstream barriers by automating transformations of free text or agency-specific vocabulary to standardized ontology terms [17, 18]. Future development of the DataHarmonizer includes integration of LexMapr (matches free text to ontology terms) and other new tools to classify and further structure information according to field type (sorting of terms into appropriate fields within the DataHarmonizer) with the goal of creating an end-to-end pipeline for data harmonization. Such a pipeline would be useful for standardizing agency-specific data being exported and shared (publicly or with trusted partners), as well as standardizing incoming data retrieved from public repositories so that it can be more easily incorporated into local analyses. The benefits of these tools are that organizations would not have to radically change their practices or infrastructure to better enable exchange and improve the quality of publicly shared data, but rather would simply need to implement the software.

Rich, well-structured contextual data are critical for pathogen surveillance, public health decision-making, as well as research and innovation. The DataHarmonizer is an important part of the data ecosystem that will empower the use of public health genomics beyond the current COVID-19 pandemic.
